# Peer Victimization and Aggressive Behavior Among Chinese Adolescents: Delinquent Peer Affiliation as a Mediator and Parental Knowledge as a Moderator

**DOI:** 10.3389/fpsyg.2018.01036

**Published:** 2018-06-28

**Authors:** Shuang Lin, Chengfu Yu, Weiqi Chen, Yunlong Tian, Wei Zhang

**Affiliations:** ^1^School of Education, Guangzhou University, Guangzhou, China; ^2^School of Management, Guangzhou University, Guangzhou, China; ^3^School of Psychology, South China Normal University, Guangzhou, China

**Keywords:** peer victimization, aggressive behavior, delinquent peer affiliation, parental knowledge, adolescent development

## Abstract

Grounded in social network theory and a risk-buffering model, this study examined whether delinquent peer affiliation mediated the association between peer victimization and adolescent aggressive behavior and whether this mediating process was moderated by parental knowledge. A total of 4,209 Chinese adolescents (48.47% male, *M*_age_ = 13.68) completed questionnaires on peer victimization, parental knowledge, delinquent peer affiliation, and adolescent aggressive behavior. Path analyses showed that delinquent peer affiliation partially mediated the relationship between peer victimization and aggressive behavior. Moreover, parental knowledge had a protective effect of buffering the adverse influence of peer victimization on aggressive behavior. This indirect link was stronger for adolescents with low parental knowledge than for those with high parental knowledge. This finding highlights delinquent peer affiliation as a potential link between peer victimization and aggressive behavior and provides an effective intervention for addressing the adverse effects of peer victimization.

## Introduction

Peer victimization among adolescents is a serious social concern, and the incidence rates are growing globally (Sullivan et al., 2006; Longobardi et al., 2017). Peer victimization is defined as a repeatedly experienced form of aggressive behavior, perpetrated within the peer group (Hunter et al., 2007). Research has shown that 9–32% of adolescents in China and other countries experienced peer victimization (Huang et al., 2013; Jackson et al., 2016; Pouwels et al., 2016). Ample research evidence has indicated that peer victimization is associated with a variety of negative outcomes, including poor school adjustment, and internalizing (e.g., depression) and externalizing problems (e.g., aggressive behavior; Longobardi et al., 2017; Noret et al., 2018). Therefore, understanding the mechanisms of peer victimization on adolescent development is essential for the development of protective intervention programs.

Adolescents spend a notable amount of time in school and with their peers, and the influence of school and peer context on adolescent development has received increased attention in recent years (Huang et al., 2013; Longobardi et al., 2017; Noret et al., 2018). Survey research show that school violence is common and has been increasing in China (Chi et al., 2010). Moreover, a climate of school violence, poor student-teacher relationships, and low social status are significant risk factors for victimization (Longobardi et al., 2018). Furthermore, Chinese adolescents may experience physical, relational (e.g., social ostracism), and verbal (e.g., being teased) victimization (Huang et al., 2013; Li et al., 2015). Ample research evidence has repeatedly shown that peer victimization is a powerful risk factor for adolescent aggressive behavior (Sullivan et al., 2006; Aceves and Cookston, 2007; Ostrov, 2010). Aggressive behavior refers to behavior that is harmful to others, with physical, verbal, and relational aggression are the most common types (Ettekal and Ladd, 2017). For instance, Sullivan et al. (2006) discovered that peer victimization was positively related to physical, verbal, and relational aggression among urban middle school students. Similarly, Ostrov (2010) demonstrated that peer victimization could predict the growth of relational aggression over time. Longitudinal research suggested a potential causal relationship between peer victimization and aggression, in which peer victimization serves as an antecedent to put adolescents at risk for physical aggressive behavior (Aceves and Cookston, 2007). These findings highlight that peer victimization is a crucial risk factor for adolescent aggressive behavior. However, its mediating and moderating mechanisms remain largely unknown. A clearer understanding of the mechanisms is important to develop an effective intervention for addressing the adverse effects of peer victimization. In this study, we aim to examine two questions: (1) whether peer victimization increases delinquent peer affiliation, which in turn increases the likelihood of adolescent aggressive behavior; (2) whether this indirect association is moderated by parental knowledge.

### Delinquent Peer Affiliation as a Mediator

Delinquent peer affiliation is one of the potential factors that links peer victimization to adolescent aggression. Delinquent peer affiliation refers to adolescent affiliation with peers who engage in deviant behaviors such as fighting, stealing, and alcohol use (Rudolph et al., 2014; Zhu et al., 2015). Based on social network theory (Veenstra and Dijkstra, 2011), adolescents affiliate with delinquent peers via different processes through homophily selection (Reynolds and Crea, 2015). Homophily selection occurs when adolescents actively affiliate with delinquent peers because of perceived similarity with them. Grounded in this perspective, victimized adolescents may voluntarily affiliate with delinquent peers who have similar experiences such as rejection, depression, and social helplessness (Dishion et al., 2012; Rudolph et al., 2014). Victimized adolescents may experience more rejection and social isolation by mainstream peers and undergo more rejection than non-victims (Dishion et al., 2012). Rudolph et al. (2014) revealed that loneliness and social helplessness facilitate subsequent affiliation with delinquent peers through homophily selection. Another explanation is that youths who are victimized may affiliate with delinquent peers via the process of default selection (Sijtsema et al., 2010). Default selection occurs when adolescents actively affiliate with peers because of their lack of viable alternatives. Victimized adolescents have lower peer acceptance because of gradual rejection by mainstream peers, and therefore, are more likely to affiliate with delinquent peers (Kochel et al., 2012). Gifford-Smith and Brownell (2003) found that victims might expect that some traits (e.g., aggressive behavior) perceived in delinquent peers can protect them from victimization. Overall, we theorized that targets of victimization are gradually forced out of traditional peer groups so that they are more likely to associate with delinquent peers.

Furthermore, delinquent peer affiliation may facilitate aggressive behavior through social learning mechanisms. Delinquent behaviors are based on attitudes and beliefs acquired through adolescents’ observational learning and imitation of delinquent peers (Bandura, 1978). Adolescents who affiliate with delinquent peers gradually urge adolescents who were exposed to peer victimization to develop aggression by repeated peer reinforcement for aggressive behavior and peer pressure to imitate others’ aggressive behavior (Dishion et al., 1996). Indeed, previous research revealed that delinquent peer affiliation mediates the association between ecological risks (e.g., maladaptive parenting, corporal punishment, poor school climate) and aggression (Laible et al., 2016; Wang et al., 2017; Zhu et al., 2017). For instance, Laible et al. (2016) reported that maternal sensitivity and temperament in early childhood could exert an indirect effect on adolescents’ aggressive behaviors via delinquent peer affiliation. Moreover, Wang et al. (2017) showed that poor school climate increased aggressive behaviors through changes in delinquent peer affiliation. Based on these arguments, we propose the following hypothesis:

Hypothesis 1: Delinquent peer affiliation will mediate the relationship between peer victimization and aggressive behavior.

### Parental Knowledge as a Moderator

Although peer victimization poses a considerable risk for adolescent aggressive behavior and delinquent peer affiliation, adolescents have resilient outcomes when subjected to environmental risk. Therefore, protective factors should also be considered when examining the association between peer victimization and aggressive behavior. Parental knowledge refers to a range of related parenting behaviors concerning attention to and tracking of their adolescent’s whereabouts, activities, and friends (Dishion and McMahon, 1998), which has received increased attention as a protective factor that can buffer the risk effects of peer adversity (Jiang et al., 2016). According to the risk-buffering model, a favorable family context such as parental knowledge can attenuate the relation between environmental risk factors (e.g., peer victimization) and problem behaviors (e.g., aggression; Luthar et al., 2015).

Parental knowledge refers to the degree to which parents are informed about their adolescents’ daily experiences (Marceau et al., 2015). Previous literature revealed that adolescents with poor parental knowledge were more likely to affiliate with delinquent peers compared to adolescents with adequate parental monitoring of trying alcohol and cigarettes (Westling et al., 2008). Meanwhile, Jiang et al. (2016) found that adolescents with inadequate parental knowledge had more delinquent peer affiliation if they experienced more peer victimization. According to the risk-buffering model, we examined whether parental knowledge moderated the indirect associations between peer victimization and aggressive behavior.

The present study is the first to test whether parental knowledge plays a moderating role between peer victimization and aggressive behavior. Dishion et al. (2002) reported that parental knowledge was associated with a reduced likelihood of delinquent peer affiliation for high-risk adolescents. Moreover, previous research has shown that parental knowledge could buffer the risk effects of peer adversity on delinquent peer affiliation (Schofield et al., 2015). Based on the risk-buffering model, it is reasonable to expect that parental knowledge could moderate the association between peer victimization and delinquent peer affiliation, which, in turn, facilitates adolescent aggressive behavior. Based on the above theoretical analyses and empirical evidence, we propose the following hypothesis:

Hypothesis 2: Parental knowledge will moderate the indirect positive association between peer victimization and adolescent aggressive behavior. This indirect association will be significant among adolescents with low parental knowledge but significantly weaker among adolescents with high parental knowledge.

### The Present Study

**FIGURE 1:**
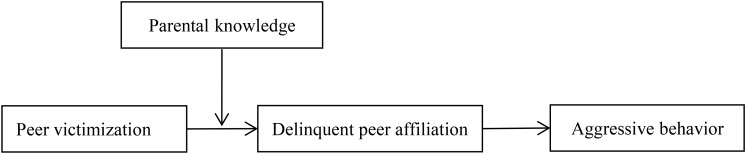
The proposed moderated mediation model.

Based on social network theory and the risk-buffering model, this study included delinquent peer affiliation as an environmental risk factor and parental knowledge as a family context factor to construct a moderated mediation model to examine the underlying mechanisms of the relationship between peer victimization and adolescent aggressive behavior. **Figure 1** illustrates the proposed research model.

## Materials and Methods

### Participant

Participants were recruited from 12 middle schools in southern China through stratified and random cluster sampling. A total of 4,209 adolescents (48.47% male) ranging in age from 10 to 19 (*M*_age_ = 13.68, *SD* = 2.75) participated in this study.

### Measures

#### Peer Victimization

Peer victimization was measured by using a 9-item Chinese version of a questionnaire used in Zhu et al.’s (2016) research. Respondents were asked to indicate their experiences of peer victimization in the past 6 months (e.g., “How many times did you get hit or kicked by peers in the past 6 months?”; “How many times were you completely ignored by peers in the past 6 months?”). All items were rated on a 5-point scale from 1 (*never*) to 5 (*six or more times*). The mean of the nine items was calculated, with higher scores reflecting higher levels of peer victimization. The Cronbach’s α coefficient was 0.87 for this questionnaire.

#### Aggressive Behavior

Aggression behavior was measured using the Chinese version of the Buss–Warren aggression questionnaire (BWAQ; Maxwell, 2008). The adolescents were asked to respond to 19 items by indicating the level of physical, relational, and verbal aggression in the past 6 months (e.g., “Given enough provocation, I may hit another person.”; “I can’t help getting into arguments when people disagree with me.”). Participants rated how well each item described themselves using the original 5-point scale, ranging from 1 (*not at all*) to 5 (*absolutely like me*). The responses were averaged across the 19 items, with higher scores indicating higher levels of aggression. The Cronbach’s α coefficient was 0.87 for this questionnaire.

#### Delinquent Peer Affiliation

We used the 12-item Chinese version of a questionnaire from a self-report survey (Zhu et al., 2015) to assess the extent of delinquent peer affiliation. Adolescents reported how many of their friends had engaged in 12 different deviant behaviors in the past 6 months (e.g., “During the past 6 months, how many of your friends engaged in fights?”). All items were rated on a 5-point scale from 1 (*never*) to 5 (*six or more*). The mean of the 12 items was calculated, with higher scores reflecting higher levels of deviant peer affiliation. The Cronbach’s α coefficient was 0.85 for this questionnaire.

#### Parental Knowledge

We used the 5-item version of the parental knowledge questionnaire used in Jiang et al.’s (2016) research, which demonstrated reliability and validity in previous studies. Adolescent respondents were asked to report the level of parental knowledge of their activities in leisure time and other periods in the past 6 months (e.g., “Do your parents know what you do during your free time?”; “Do your parents know where you go when you are out with friends at night?”). This questionnaire rated items on a 3-point scale ranging from 1 (*know little*) to 3 (*know a lot*). Means were calculated for all items; higher scores reflect higher levels of parental knowledge. The Cronbach’s α coefficient was 0.78 for this questionnaire.

### Procedures

Permission to conduct the study was granted by the research ethics committee of the author’s university. Written informed consent was obtained from the teachers of the participating schools, as well as all adult participants and the parents/legal guardians of adolescent participants prior to beginning data collection. Participants were invited to participate in their classrooms during class time. Trained teachers handed out the self-report questionnaires to adolescents in their classrooms. After a complete description of the study, all student participants were told that their responses would be kept strictly confidential and that their participation was voluntary. Participants were also told that they should complete the questionnaire independently. To encourage honest reporting, adolescents were given approximately 30 min to complete the anonymous questionnaires.

### Statistical Analysis

SPSS 20.0 was utilized for descriptive statistics. We used Mplus 7.1 (Muthén and Muthén, 1998/2012) to perform structural equation modeling with the full-information maximum likelihood estimation method to examine mediation and moderation effects. Model fit was assessed using three indices: (1) the chi-square statistic and normed chi-square (*χ*^2^/*df*), (2) comparative fit index (CFI), and (3) root mean square error of approximation (RMSEA). Model fit is considered to be excellent when *χ*^2^/*df* ≤ 3, CFI ≥ 0.95, and RMSEA ≤ 0.06 (Kline, 2011; Hoyle, 2012). In addition, adolescents’ gender, age, and impulsivity were controlled in statistical analyses because previous research has revealed that these demographic factors are associated with aggressive behavior (Pérez Fuentes et al., 2016).

## Results

### Preliminary Analyses

**Table 1** displays the means and standard deviations of the variables representing peer victimization, delinquent peer affiliation, parental knowledge, aggressive behavior, and correlations for all variables in the current study. The results showed that peer victimization was significantly and positively associated with aggressive behavior, which indicated that higher levels of aggression were associated with higher levels of peer victimization. In addition, parental knowledge was negatively associated with delinquent peer affiliation and aggressive behavior, whereas delinquent peer affiliation was positively associated with aggressive behavior.

**Table 1:** Means, standard deviations, and correlations of the main study variables.

Variables	1	2	3	4	5	6	7
(1) Gender	1.00						
(2) Age	−0.04**	1.00					
(3) Impulsivity	0.02	0.19**	1.00				
(4) Peer victimization	0.05**	−0.01	0.24**	1.00			
(5) Parental knowledge	−0.11**	−0.24**	−0.27**	−0.11**	1.00		
(6) Delinquent peer affiliation	0.14**	0.13**	0.22**	0.34**	−0.19**	1.00	
(7) Aggressive behavior	0.12**	0.13**	0.35**	0.30**	−0.13**	0.26**	1.00
*M*	0.48	13.68	2.26	1.51	2.40	1.28	1.73
*SD*	0.50	2.75	0.38	0.72	0.51	0.47	0.53

*Gender was dummy coded by 1 = male and 0 = female. ^∗^p < 0.05. ^∗∗^p < 0.01.*

### Testing for a Mediation Effect

**FIGURE 2:**
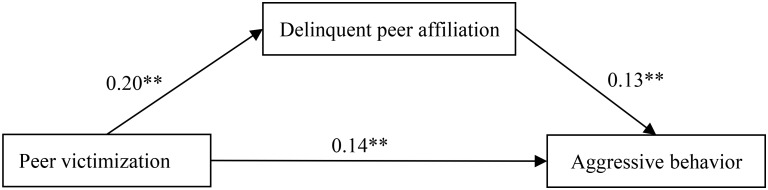
Model of the mediating role of delinquent peer affiliation between peer victimization and aggressive behavior. Non-significant paths, and paths between gender, age, impulsivity, and each of the variables in the model are not displayed. Of those paths, the following were significant: gender, age and impulsivity to delinquent peer affiliation (*b*_1_ = 0.12^∗∗^, *b*_2_ = 0.02^∗∗^, *b*_3_ = 0.15^∗∗^) and aggressive behavior (*b*_1_ = 0.10^∗∗^, *b*_2_ = 0.01^∗∗^, *b*_3_ = 0.36^∗∗^). ^∗^*p* < 0.05,^∗∗^*p* < 0.01.

The mediation model revealed that the model is identified to the data: *χ^2^*/*df* = 0.000, CFI = 1.000, RMSEA = 0.000. The results are displayed in **Figure 2**. Peer victimization positively predicted delinquent peer affiliation (*b* = 0.20, *SE* = 0.01, *t* = 20.78, *p* < 0.01) and positively predicted aggressive behavior (*b* = 0.14, *SE* = 0.01, *t* = 12.86, *p* < 0.01), and delinquent peer affiliation positively predicted aggressive behavior (*b* = 0.12, *SE* = 0.02, *t* = 7.63, *p* < 0.01). Moreover, bootstrapping analyses indicated that delinquent peer affiliation partially mediated the relation between peer victimization and aggressive behavior [indirect effect = 0.0255, *SE* = 0.0045, 95% CI (0.0167, 0.0344)].

### Testing for Moderated Mediation

**FIGURE 3:**
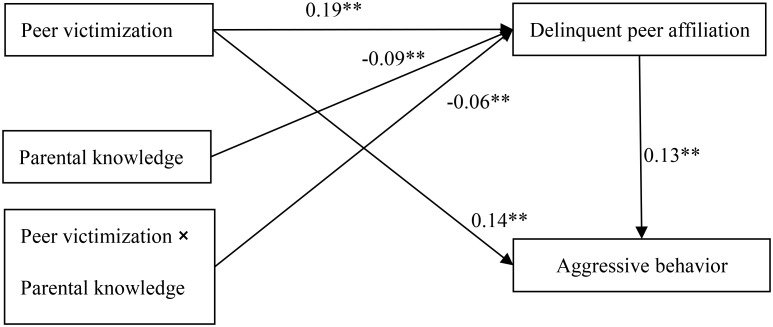
Model of the moderating role of parental knowledge on the indirect relationship between peer victimization and aggressive behavior. Non-significant paths, and paths between gender, age, impulsivity, and each of the variables in the model are not displayed. Of those paths, the following were significant: gender, age, and impulsivity to delinquent peer affiliation (*b*_1_ = 0.11^∗∗^, *b*_2_ = 0.02^∗∗^, *b*_3_ = 0.13^∗∗^) and aggressive behavior (*b*_1_ = 0.10^∗∗^, *b*_2_ = 0.01^∗∗^, *b*_3_ = 0.36^∗∗^). ^∗^*p* < 0.05,^∗∗^*p* < 0.01.

**FIGURE 4:**
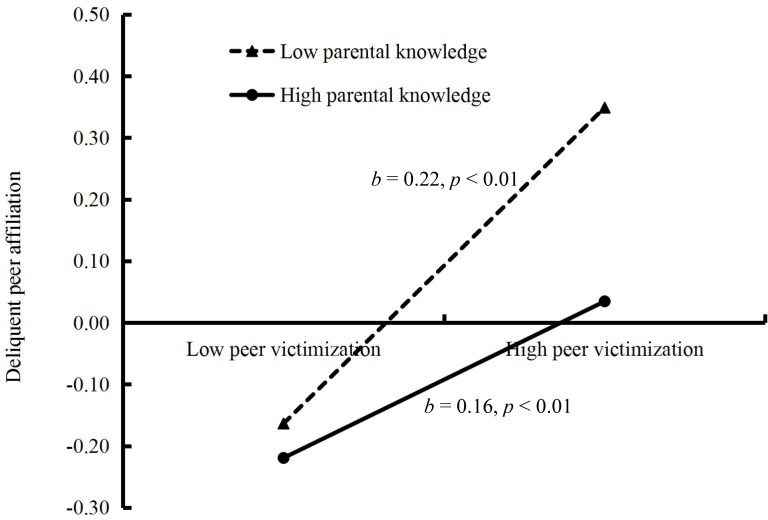
Results from the test of simple slopes to assess the association between peer victimization and delinquent peer affiliation at high and low levels (*M* ±*SD*) of parental knowledge.

The moderated mediation model represented in **Figure 3** had an excellent fit to the data: *χ*^2^/*df* = 1.066, CFI = 1.000, RMSEA = 0.004. The results showed that: (1) Parental knowledge moderated the association between peer victimization and delinquent peer affiliation (*b* = -0.06, *SE* = 0.02, *t* = -3.68, *p* < 0.01). We conducted simple slope plots and calculations at -1 *SD* and +1 *SD* from the mean of peer victimization and as depicted in **Figure 4**. The positive association between peer victimization and delinquent peer affiliation was much stronger for adolescents with lower parental knowledge (*b* = 0.22, *SE* = 0.01, *t* = -18.07, *p* < 0.01) compared to adolescents with higher parental knowledge (*b* = 0.16, *SE* = 0.01, *t* = 11.62, *p* < 0.01). (2) Moreover, peer victimization was negatively linked to aggressive behavior (*b* = 0.14, *SE* = 0.01, *t* = 12.86, *p* < 0.01). Additionally, delinquent peer affiliation was positively linked to aggressive behavior (*b* = 0.19, *SE* = 0.02, *t* = 10.66, *p* < 0.01). However, there was no significant interaction between parental knowledge and delinquent peer affiliation to predict aggressive behavior (*b* = -0.04, *SE* = 0.03, *t* = -1.36, *p* > 0.05).

Results revealed that conditional indirect effects were found to be significant for adolescents with lower parental knowledge [indirect effect = 0.0309, *SE* = 0.0061, 95% CI (0.0184, 0.0430)] and for adolescents with higher parental knowledge (indirect effect = 0.0098, *SE* = 0.0053, 95% CI [0.0003, 0.0211]). Thus, adolescents with lower parental knowledge were more likely to associate with delinquent peers, which in turn contributed to higher levels of aggressive behavior.

## Discussion

Burgeoning evidence for the adverse impacts of peer victimization on children’s adjustment has garnered considerable empirical support (Rudolph et al., 2014; Jiang et al., 2016; Zhu et al., 2016). However, little research has examined mediating and moderating mechanisms of this association to demonstrate how and for whom peer victimization impacts adolescent aggressive behavior. The current study sought to address this gap by formulating a moderated mediation model to examine whether peer victimization was indirectly related to adolescent aggressive behavior through increased delinquent peer affiliation and whether parental knowledge moderated this indirect association.

### The Mediating Role of Delinquent Peer Affiliation

The current study is the first to document how delinquent peer affiliation functions as a mediator in the association between peer victimization and aggressive behavior, which was consistent with our first hypothesis. When youth are exposed to peer victimization, they are more likely to affiliate with delinquent peers, which in turn increases their aggressive behavior. This finding is congruent with social network theory and social learning mechanisms. The indirect effects of peer victimization on aggressive behavior were mediated by delinquent peer affiliation, which was congruent with prior studies showing that delinquent peer affiliation mediated peer context influences on adolescent internalizing and externalizing problems (Chen et al., 2015; Zhu et al., 2016).

Peer victimization is a serious, general issue, and may facilitate multiple adverse adolescent developmental outcomes. Based on the perspective of social network theory, this research can explain the association between peer victimization and delinquent peer affiliation. Victimized children may be treated negatively and be avoided by their peers (Kochel et al., 2012). As a result, compared to non-victims, targets of victimization would be more disliked and more likely to be alienated from the mainstream peer group. Moreover, victimized children often feel alone, excluded, and dissatisfied by their peer relationships (Rudolph et al., 2014). In turn, victimized children who are rejected by mainstream peers and who feel alone may try to improve their situation by seeking affiliations with delinquent peers through a default selection process, or they may passively seek out these affiliations to meet basic psychological needs (Sijtsema et al., 2010).

Furthermore, we found that delinquent peer affiliation was positively associated with aggressive behavior. This finding is consistent with previous research showing that peer relations are a risk factor for problem behaviors (Zhu et al., 2015; Laible et al., 2016). From a social learning perspective, which can explain this association, adolescents who affiliate with delinquent peer may actively develop aggressive behaviors because of their frequent contact, shared activities, and interpersonal affective connectedness with delinquent peers. Therefore, they may actively imitate aggressive behaviors (Dishion et al., 1996), especially in the context of Chinese culture, which places greater emphasis on youth having a collectivist orientation (Chen, 2000). Moreover, aggressive behaviors seem to be increasingly reinforced via social benefits in the delinquent peer group.

### The Moderating Role of Parental Knowledge

As we expected, this study found that parental knowledge played a moderating role in the indirect relationship between peer victimization and aggressive behaviors via delinquent peer affiliation. Specifically, parental knowledge moderated the direct effect of peer victimization on delinquent peer affiliation. Combined with the risk-buffering model, a simple slopes analysis of the interaction showed that adequate parental knowledge might be a risk-buffering factor for adolescent problem behavior (Ang et al., 2012), protecting adolescents from delinquent peer affiliation even if they are the targets of peer victimization. Moreover, peer victimization was associated with less delinquent peer affiliation under the condition of higher levels of parental knowledge, but more delinquent peer affiliation under the condition of lower levels of parental knowledge. Adolescents with adequate parental knowledge, in which parents of adolescents keep track of their adolescents’ whereabouts and activities, can easily obtain support from their parents to deal with adversities when their parents know that their children are experiencing peer victimization at school. In addition, adequate parental knowledge provides communication and the context for open parent-adolescent relationships, which are important in facilitating adolescents’ positive adjustment in adverse contexts (e.g., peer victimization).

On the other hand, as we expected, parental knowledge played a moderating role and was better able to differentiate levels of aggressive behaviors at higher rather than lower levels of delinquent peer affiliation. Specifically, the moderating effect of low parental knowledge was stronger than that of high parental knowledge. Our findings expand previous empirical research and theories by demonstrating that parental knowledge is an effective parenting strategy for decreasing adolescents’ aggressive behaviors, which is consistent with previous research emphasizing that parent-adolescent relationships are important for adolescent externalizing behavior (Dishion and McMahon, 1998; Marceau et al., 2015). Parental knowledge stems in part from adolescent disclosure of information on their whereabouts and activities, which can exert an environmental influence serving to reduce adolescent externalizing (Marceau et al., 2015). Another potential explanation is that adolescents have fewer opportunities to engage in aggressive behavior when parents are aware of their whereabouts and activities. This finding suggests that enhancing the level of parental knowledge is an effective method for interventions aiming to prevent adolescent aggressive behaviors.

In sum, the results supported our hypothesis that parental knowledge moderates the indirect relationship between peer victimization and aggressive behaviors. The finding revealed that parental knowledge as a protective factor mitigates the adverse impact of peer victimization on aggressive behaviors during early adolescence. This finding suggests that the mechanisms underlying associations between parenting and adolescent behavior will be important for informing interventions both in terms of reducing negative behavior and in terms of promoting positive development by identifying optimal targets of intervention.

### Limitations and Future Directions

The findings in the current study should be interpreted in the context of several limitations. First, the study cannot establish causality among variables because of the cross-sectional design. Specifically, previous research has found a link between delinquent peer affiliation and aggressive behaviors, which could be bidirectional (Werner and Crick, 2004). Therefore, future research should use longitudinal designs to delineate the directionality of these relationships better. Second, because our study data were based on adolescents’ self-reports, the results may have limited generalizability due to biases in reporting. Nonetheless, self-reports of peer victimization (Jiang et al., 2016) and delinquent peer affiliation (Fergusson and Horwood, 1999) can provide valuable information. For example, Stattin and Kerr (2000) found that assessing parental knowledge by youth self-report may be more predictive of adolescent aggressive behaviors than parental report, as adolescents have a better sense of the true extent of their parents’ knowledge. Future studies should utilize multiple methods (e.g., peer and child reports) and multiple informants to assess the variables of home and peer contexts. Third, the study was conducted in the context of Chinese culture; therefore, we must treat our results with caution when extending them to different cultural or demographic groups. Finally, although the overall sample size was relatively large, there are concerns about the generalizability of our findings, as it is not clear whether our findings could be generalized to clinical samples. Moreover, we are also not clear that our findings have universal applicability to related outcomes (e.g., depression, suicidal behavior) of more specific domains of aggression (e.g., physical aggression, language aggression).

## Author Contributions

CY and SL designed the work. CY, SL, and YT collected the data. SL, CY, and WZ analyzed the data results and drafted the manuscript. CY, WC, and WZ revised the manuscript. CY and WZ approved the final version to be published.

## Conflict of Interest Statement

The authors declare that the research was conducted in the absence of any commercial or financial relationships that could be construed as a potential conflict of interest.
